# Inconvenient Samples: Modeling Biases Related to Parental Consent by Coupling Observational and Experimental Results

**DOI:** 10.1162/opmi_a_00031

**Published:** 2020-03-01

**Authors:** Yue Yu, Patrick Shafto, Elizabeth Bonawitz

**Affiliations:** Centre for Research in Child Development, National Institute of Education, Singapore; Rutgers University-Newark; Rutgers University-Newark

**Keywords:** parental consent, parent–child interaction, pedagogical question, multiple imputation, propensity score matching

## Abstract

In studies involving human subjects, voluntary participation may lead to sampling bias, thus limiting the generalizability of findings. This effect may be especially pronounced in developmental studies, where parents serve as both the primary environmental input and decision maker of whether their child participates in a study. We present a novel empirical and modeling approach to estimate how parental consent may bias measurements of children’s behavior. Specifically, we coupled naturalistic observations of parent–child interactions in public spaces with a behavioral test with children, and used modeling methods to impute the behavior of children who did not participate. Results showed that parents’ tendency to use questions to teach was associated with both children’s behavior in the test and parents’ tendency to participate. Exploiting these associations with a model-based multiple imputation and a propensity score–matching procedure, we estimated that the means of the participating and not-participating groups could differ as much as 0.23 standard deviations for the test measurements, and standard deviations themselves are likely underestimated. These results suggest that ignoring factors associated with consent may lead to systematic biases when generalizing beyond lab samples, and the proposed general approach provides a way to estimate these biases in future research.

## INTRODUCTION

Sampling and generalizability are the methodological bedrocks of behavioral science, and knowing whether the sample is representative of the population is critical to the validity and generalizability of research findings. Among the many factors that may bias the sampling process, one prevalent but understudied factor is the refusal to participate in research. The goal of this study is to develop a method to estimate would-be experimental performance for those who did not consent, and to inform the generalizability of research findings.

We chose to focus on one of the fields in which behavior tends to be heterogeneous along factors that may associate with nonenrollment: research with young children. Before the start of schooling, children’s experiences are heavily influenced by the values and practices of their parents, which are known to be heterogeneous both within and between social groups (Bornstein, 1991; Hoff, Laursen, Tardif, & Bornstein, 2002). Yet, parents are also the ones who decide whether their children participate in research, and those same values and practices may play a role in their decision to consent. If variables that influence the likelihood of parental consent also influence children’s behavior, then this presents a major hurdle for generalizing findings from the field.

Nonenrollment may also have larger impacts on fields in which substantial proportions of potential participants decline or ignore recruitment efforts from researchers. Through a survey among lab managers and project leaders, we estimated the base rate of parental consent in developmental experiments as approximately 50%, which also differed based on recruitment methods (see Supplemental Materials A; Yu, Shafto, & Bonawitz, 2020). This suggests a substantial rate of nonenrollment, which necessitates closer examination of its impact.

To date, little is known about whether and how parental consent may bias findings from experiments with young children. We know that in the field of survey-based research with school-aged children and adolescents, parental consent has been associated with family demographics and student behavior (Kearney, Hopkins, Mauss, & Weisheit, 1983; Lueptow, Mueller, Hammes, & Master, 1977). Moreover, students recruited through passive consent (which requires a reply to opt out of a study) differ from students recruited through active consent (which requires a reply to opt in) in a number of characteristics, including race, family environment, school performance, and percentage of at-risk youth (Anderman et al., 1995; Dent et al., 1993; Esbensen, Miller, Taylor, He, & Freng, 1999). However, this type of research is lacking regarding children before school age, possibly due to a lack of archival information like school records, as well as ethical concerns in using passive consent for experiments that require face-to-face interactions with young children. More importantly, although existing research on parental consent has shed light on *who* is underrepresented because of nonenrollment, we still know little about *whether* and *how* nonenrollment may bias research findings.

Existing research on nonenrollment also tends to focus on studies with correlational designs, but experimental research is subject to biases related to nonenrollment as well. Whereas randomly assigning participants to treatment and control groups may eliminate systematic between-group differences on potential confounding factors, the effects of treatment found in such studies are still confined by the characteristics of the research sample *before* the random assignment, and may not apply to those who are underrepresented in the sample from the start.

This study takes a first step to investigate whether and how factors associated with parental consent affect research findings in developmental experiments. We developed a novel approach to achieve that goal: coupling naturalistic observations of public parent–child interactions with behavioral tests with children. Observations of public behavior are commonly used in sociology, anthropology, and psychology to study human behavior (Goffman, 1966). Specific to developmental psychology, researchers have observed and live-coded children’s and adults’ actions and interactions in public spaces like zoos and supermarkets (Ridge, Weisberg, Ilgaz, Hirsh-Pasek, & Golinkoff, 2015; Whiten et al., 2016), and these studies have contributed to our understanding of people’s naturalistic behavior without awareness of being in an experiment.

We started with observing parent–child interactions in public spaces to obtain a relatively representative distribution that is unaffected by the consent process. During the observation, we coded aspects of parent–child interactions that are known to be causally linked to children’s behavior. We then invited children who were observed to participate in a behavioral test. By analyzing the correlations between the observational and test data, and between the observational data and participation, we looked for predictors that may associate test data with participation itself, which would indicate a difference in test data between children who participated and did not participate. We then used model-based multiple imputation and propensity score–matching procedures to simulate the behavior of children who did not participate.^1^ Finally, these simulation results were used to assess whether the means and standard deviations of the participating group were biased estimations for those of the initial population.

We examined a domain where there is known heterogeneity in parenting practices: asking questions to teach. This line of research is grounded in a rich literature about informal pedagogy (Bonawitz et al., 2011; Csibra & Gergely, 2009), which suggests that the format in which parents and educators choose to present evidence to children influences how children infer and learn. Specifically, recent experiments have shown that questions asked by knowledgeable adults improve children’s learning (Gutwill & Allen, 2010, 2012; Haden, Cohen, Uttal, & Marcus, 2015), and these “pedagogical questions” are particularly effective in facilitating children’s exploratory learning of causal properties of a novel artifact (Yu, Landrum, Bonawitz, & Shafto, 2018). Therefore, in this study we examine whether asking pedagogical questions may also be associated with parental consent, thus leading to biases in measurements of children’s exploratory learning. We examined this hypothesis by replicating one condition of the previous experiment (Yu et al., 2018), in which an experimenter asked a pedagogical question about a novel toy before leaving children to explore that toy. Here we added a critical observation phase, in which parents’ pedagogical questions toward children were coded along with other parent–child interaction measurements. This allows us to look for associations between parents’ pedagogical questions and children’s exploratory learning in the test phase. And because the observational data are available for children who did not participate, these associations could then be used to simulate what the not-participating children would have done in the test. The final goal is to compare the results from the participating group and the results we would have obtained if all parents consented. A shortened version of this research was presented in the *Proceedings of the 39th Annual Conference of the Cognitive Science Society* (Yu, Bonawitz, & Shafto, 2017). This report includes results from new analyses and a new survey, which resulted in a more robust and grounded method of evaluating biases related to nonparticipation.

## METHOD

### Participants

We set up the study in two sites: an indoor reptile exhibit in a zoo, and an indoor playground. We chose these two sites to ensure diversity in the population we initially observed (for details see Supplemental Materials B; Yu et al., 2020). Seventy-eight parent–child dyads (41 from the zoo and 37 from the playground) were observed and then invited for the test. All children were between 3 and 6 years of age. Thirty-one additional dyads were observed but were not invited for the test, for reasons detailed in the Supplemental Materials B (Yu et al., 2020).

### Procedure

This study was approved by the Internal Review Board of Rutgers University-Newark. The observation phase was considered observation of public behavior based on the guidelines from the Department of Health and Human Services, and therefore exempted from the requirement of obtaining informed consent. During each trip to the test sites, three researchers collected data from parent–child dyads in three phases: Two coders first observed and coded the interactions between the parent and the child (observation phase). Then a third researcher invited the dyad to participate in a test (recruitment phase). She and one of the coders conducted the test if the dyad agreed to participate (test phase).^2^

#### Observation Phase.

Two coders who were blind to the study hypotheses pretended to be visitors so that they could code parent–child interactions without the dyad’s awareness. Each dyad was observed for 5 minutes, during which the coders independently coded the length of parent–child interactions and the frequency of parent–child communications. Length of parent–child interactions was measured by the time period of dyadic activities (parent and child engaging in the same activity), supervised activities (parent watching, following, or taking pictures of child when child is engaging in his or her own activities), and unsupervised activities (parent and child engaging in different activities). Frequency of parent–child communications was measured using an adaptation of the Dyadic Parent–Child Interaction Coding System (Eyberg, Nelson, Ginn, Bhuiyan, & Boggs, 2013): The coders recorded the numbers of parents’ questions, statements, and commands toward children. Critical to our interest, parents’ questions were further differentiated based on their functions (Yu, Bonawitz, & Shafto, 2016): Those used to help children learn were coded as “pedagogical questions,” whereas those used to request information from children were coded as “information-seeking questions.” Interrater reliabilities were computed based on all observations, and were high across all measurements: Interrater correlation *r* = .78 ∼ .84 for the length of parent–child interactions, and *r* = .79 ∼ .86 for the frequency of parent–child communications. The average of the two coders’ codes were used for data analysis.

#### Recruitment Phase.

After the 5-minute observation, a third researcher who was blind to the observation phase approached the parent and invited the parent–child dyad to participate in a test. The recruitment procedure followed a script that resembled that of a typical developmental experiment. Among the 78 parent–child dyads that were observed, 59 agreed to participate and 19 refused. The 19 dyads who refused to participate comprised the “not-participating” group. The consent rate (75.6%) is similar to the average consent rate of onsite recruitment indicated in our survey (84.4%). Of the 59 parents who agreed, data from the test phase were available for 47 children, who comprised the “participating” group (age 3.0y to 6.3y).

#### Test Phase.

Parents and children who agreed to participate were led to a corner of the zoo exhibit or a separate room in the indoor playground, where the test was conducted by the recruiter (acting as an experimenter) and one of the coders (acting as a confederate). The materials and procedure of the test were identical to the pedagogical question condition in Yu et al. (2018), and details are included in Supplemental Materials E (Yu et al., 2020). Children were presented with a novel toy that, unbeknownst to them, has five functional parts. The experimenter explained that she knew all about the toy, then pointed to a button (which is the trigger of one of the functions) and asked, “What does this button do?” Children were then left alone to play with the toy until they stopped playing and signaled the researchers. The whole phase was video-recorded.

#### Video Coding.

After data collection, the videos from the test phase were coded by a new research assistant who was blind to the observation phase and the hypotheses of the study. She first determined the total time children spent playing with the toy, and then coded three measurements regarding both the whole playing period, and the first minute after children started playing: whether children activated the target function (the one triggered by the button), the number of unique actions they performed with the toy, and the number of nontarget functions (out of 4) they activated. A second assistant, also blind to the observation phase and study hypotheses, coded 14 (30%) of the videos for reliability. The interrater reliability agreement was high for all measurements (*r*s and *κ*s > .75; for details see Supplemental Materials F; Yu et al., 2020). To better capture individual differences in children’s exploratory learning, we further standardized all outcome variables across children and created two composite scores for each child: Exploration variability is the sum of *z*-scores of all measurements during the whole playing period. Exploration efficiency is the sum of *z*-scores of all measurements during the first minute of play.

### Data Analysis

Between-group comparisons, correlations, and regressions were conducted in IBM SPSS 22. Fisher’s exact test was used for comparisons of frequencies. Model-based multiple imputation was implemented with the Multiple Imputation module of SPSS. Bootstrapping and propensity score matching was implemented with R 3.2.3. An *α* level of .05 (two-tailed) was used for all tests.

## RESULTS

The consent rates and children’s behavior in the test did not differ significantly across test sites (for details see Supplemental Materials G; Yu et al., 2020), therefore data from the two sites were combined.

### Are Parent–Children Interactions Associated With Children’s Behavior in the Test?

**Figure 1.**
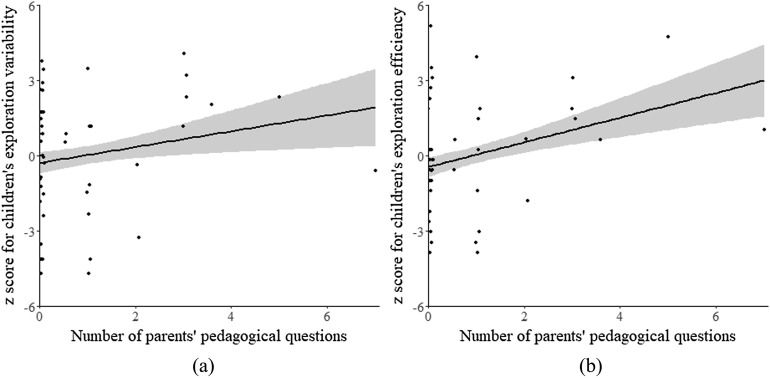
**Children whose parents asked more pedagogical questions explored more variably during the whole playing period (a), and also explored more efficiently during the first minute of play (b).** The shaded area depicts the 95% confidence interval, which means there is a 95% chance that the true linear regression line of the population will lie within the area. This is different from a 95% prediction interval, which means there is a 95% chance that the real value of *y* corresponding to certain *x* will lie within the area.

Because previous research has suggested an association between parents’ pedagogical questions and children’s exploratory learning (Yu et al., 2018), we first examined correlations between these measures. Results have confirmed our hypothesis: after controlling for test site and age, children whose parents asked more pedagogical questions received higher scores in both exploration variability and exploration efficiency, *r*s > .3, *p*s < (Figure 1). Also, children of parents who spent more time interacting with them were more efficient in their exploration, *r*(42) = .35, *p* = .021. On the other hand, measurements regarding the composition of the group being observed (parent’s and child’s gender, and whether they were accompanied by other adults or children) did not correlate with exploration variability or efficiency, *p*s >.01 (for details see Supplemental Materials H and Table S1; Yu et al., 2020). These results suggest that patterns observed in parent–child interactions were indeed associated with children’s exploratory learning during the test.

### Are Parent–Child Interactions Associated With Participation?

We fitted a logistic regression model with participation as the outcome variable and the observational measurements as predictors (Table S2; Yu et al., 2020). Overall, the predictors were able to explain a significant amount of variance in participation, Nagelkerke’s *R*^2^ = .297. Among individual predictors, we first examined the role of parental pedagogical questioning, which has been shown to be associated with children’s behavior in the test. As predicted, parents who asked more pedagogical questions during observation were also more likely to have their children participate in the test, *B* = 1.49, *p* = .047. In addition, parents were more also likely to have their boys participate than girls, *B* = 1.47, *p* = .032.

### What Can Be Predicted for Children Who Did Not Participate?

**Figure 2.**
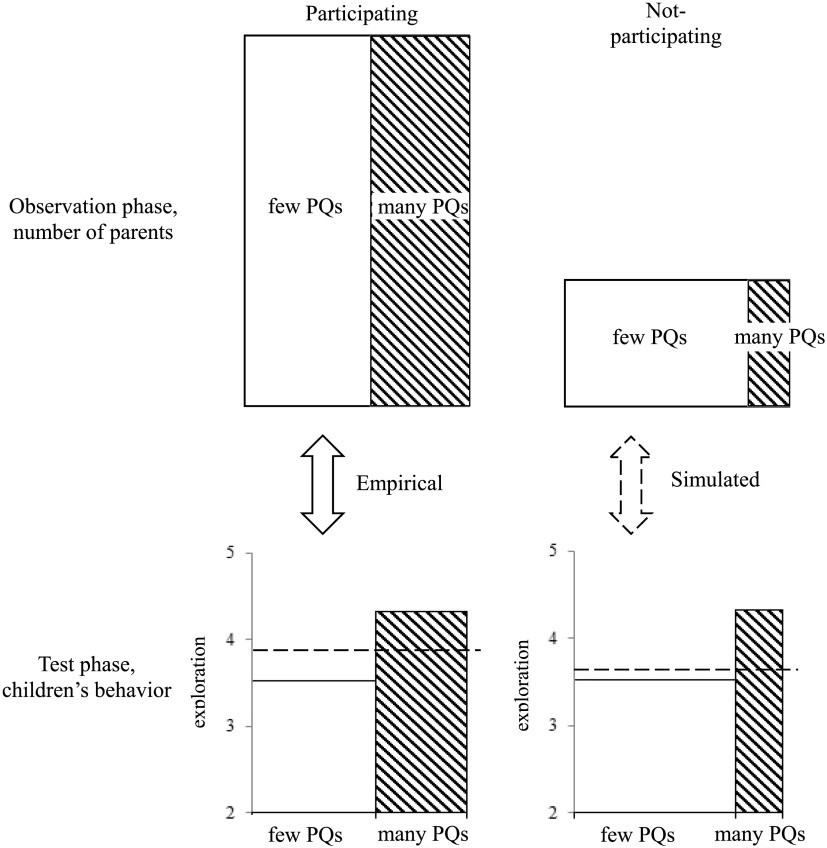
**Illustration of why nonenrollment may lead to biased estimates of test measurements.** Consider the scenario where certain parental practices, such as asking pedagogical question (PQ), is associated with both parental consent and children’s behavior in the test (e.g., exploration). In this example, we know from the observation phase that parents who asked many PQs consist of a larger proportion in the participating group (top-left) than in the not-participating group (top-right). We also know from the test phase that children of those parents (shaded bar in bottom-left) scored higher in exploration than children whose parents asked few PQs (open bar in the bottom-left). Assuming a similar relationship between PQ and exploration in the not-participating group (bottom-right), the different compositions of the two groups with regard to parental PQ will result in different averages of the test measurements: The mean estimate of exploration is lower in the simulated not-participating group (bottom-right, dashed line) than in the participating group (bottom-left, dashed line). Therefore, ignoring the not-participating group may result in an overestimation of children’s exploration.

Results so far have shown that the number of pedagogical questions parents asked children predicted both the consent for children’s participation in a test and children’s behavior during the test. This indicates that children’s participation and behavior may be related as well—that is, if we had tested children whose parents did not consent them to participate, they may have responded differently than children who did participate (Figure 2).

To test this hypothesis, we applied model-based multiple imputation to our data (Rubin, 2004). We first fitted regression models to predict the seven test measurements from the seven observational measurements, based on data from the participating group. The resulting models were then used to predict behavior of the not-participating group stochastically for 100 independent runs of simulations.^3^

Results showed that across the 100 runs of simulations, the means of the imputed not-participating groups were significantly lower than the participating group for five out of the seven test measurements (Table S3; Yu et al., 2020). When we compare the imputed groups to size-matched subsamples randomly chosen from the participating group (bootstrapping groups, also resampled for 100 runs), these mean differences reached statistical significance for all five measurements (Figure S1; Yu et al., 2020). This implies that the mean differences were robust and not caused by the stochastic nature of the imputation procedure. Furthermore, all the mean differences were in the same direction—they all suggested that the not-participating group would have learned and explored less with the toy. The effect sizes (Cohen’s *d*) of these differences ranged from 0.08 to 0.23, which means that the systematic between-group differences could be as much as 23% of the pooled within-group standard deviations. In addition to these mean differences, the standard deviations of the simulated not-participating groups are significantly higher than that of the participating group for six out of the seven measurements (Figure S1; Yu et al., 2020).

**Figure 3.**
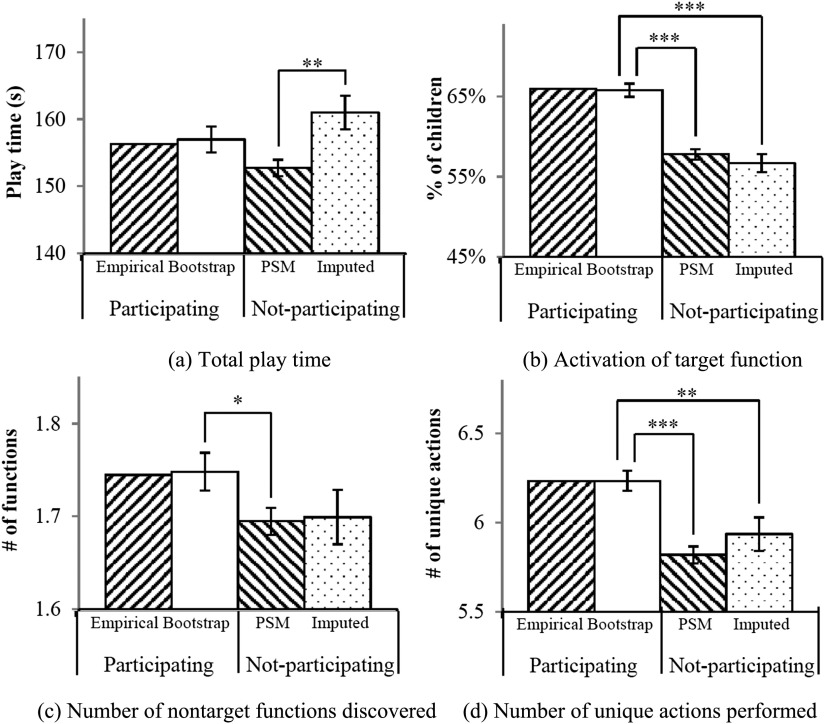
**Examples of comparisons of the estimated group means between the participating group and the simulated not-participating group.** Empirical = children who actually participated in the test phase (*n* = 47); Bootstrap = randomly selected subsamples of the participating group that matches the not-participating group in size (*n* = 19); PSM = subsamples of the participating group that matches the not-participating group in size and propensity score (*n* = 19); Imputed = simulations of the not-participating group using a model-based multiple imputation procedure (*n* = 19). To examine the robustness of simulation results, the Bootstrap, PSM, and Imputed groups were resampled for *m* = 100 runs, and the standard error across all runs are shown in the figure as the error bars. For all measurements except for total play time, the estimated group means of the not-participating group were significantly lower than that of the participating group, indicating an overestimation of children’s test performance by not considering those who were not consented to participate. Figures of all measurements can be found in the Supplemental Materials (Figure S1; Yu et al., 2020). *p < .05; **p < .01; ***p < .001.

To verify our predictions from multiple imputation, we used propensity score matching (PSM; Rosenbaum & Rubin, 1985) to select subsamples of the participating group that matched the not-participating group in size as well as the probability to participate (i.e., although these children actually participated in the test, the way their parents interacted with them during observation resembled those who did not participate, thus resulting in a low propensity score for participation).^4^ Details about the matching methods are provided in the Supplemental Materials (Yu et al., 2020). Group means of test measurements from the PSM groups resembled that of the imputed groups and not that of the participating group (Figure 3 and Figure S1; Yu et al., 2020): For six out of seven measurements, the PSM groups had significantly lower means than the bootstrapping groups, indicating that children whose parents interacted with them like those who did not participate learned and explored less with the toy. At the same time, the PSM groups did not significantly differ from the imputed groups for five out of these six measurements, which validates the imputed results.

### Are Predictions From the Models Valid?

We performed cross-validation to confirm the validity of our multiple imputation and propensity score–matching procedures. Specifically, we chose one factor that was uncorrelated to the test measurements, children’s gender, and explored whether our models could recover hypothetically “missing” data of one gender without any systematic biases.^5^ To do that, we randomly selected half of the boys who participated in the study (*n* = 18) and falsely left out their testing measurements. We then ran the multiple imputation and propensity score–matching procedures on the remaining data to recover these left-out values, and compared the results to the observed values. Results (Table S4 and Figure S2; Yu et al., 2020) showed that for six out of the seven test measurements, the observed group mean fell within the 95% confidence interval of the simulated group mean, and the effect sizes (Cohen’s *d*) of the differences between the observation and simulation are less than 0.05 for all measurements. We also observed no systematic bias for the standard deviations of the simulated group as compared to the observed group. These results suggest that the differences between the simulated not-participating group and the observed participating group are not caused by biases inherent to our models.

### What Are the Implications for Generalizing From the Participating Group to the Population?

By combining measurements from the participating group with those from the imputed not-participating group, we can estimate the means and standard deviations of the initial population during the observation phase, and then compare them to the participating group to look for potential biases. Results showed such biases to exist in the means for five out of seven test measurements, and in the standard deviations for all seven measurements (Table S3; Yu et al., 2020). Compared to the initial population, focusing on the participating group results in consistent overestimation of children’s performance in exploring the toy, as well as consistent underestimation of individual differences.

## DISCUSSION

This study takes a first step toward evaluating whether experimental findings from young children can be generalized to the population, despite nonenrollment caused by lack of parental consent. We estimated these potential biases by pairing a behavioral test with naturalistic observations of parent–child interactions prior to parental consent. Results have shown that a specific parenting practice—asking questions to teach—is correlated with both parents’ tendency to have their children participate in the test, and children’s exploratory learning during the test. And since the observational data were also available for those who did not participate, we were able to exploit these associations to simulate behavior for the not-participating children. Results from model-based multiple imputation and propensity score matching showed differences in group means between the participating and not-participating groups for five out of the seven test measurements. Furthermore, the participating group showed a lower standard deviation than the population for all test measurements.

It is worth noting that several assumptions underlie these simulated estimates. First, we assumed no direct causal relation between parents’ decisions to have their children participate and children’s potential behavior in the test, therefore the behavior of not-participating children can be considered missing at random (Rubin, 2004). This assumption is plausible because parental values and practices associated with consent, not parental consent per se, are the factors that causally influence children’s behavior. Second, our approach depends on variations in parent–child interactions for both the participating and not-participating groups, as well as a significant overlap between the two groups. This allows us to find subsamples of the participating group that matches the not-participating group in their propensity to participate, and also allows imputation to be done as interpolations within the ranges of empirical support. In short, our methods to generalize experimental results are themselves subject to usual conditions for generalization.

How much this new approach could be and should be implemented in developmental experiments would also depend on various factors. First, our approach could be beneficial for research settings that provide opportunities to observe and recruit from a relatively diverse population, such as in public spaces. Second, our approach could be more valuable for domains in which parent–child interactions and children’s behavior are expected to be associated with enrollment via common predictors. In our case, both parental questioning and children’s exploratory learning could relate to traits like curiosity, which may also predict families’ tendency to enroll in research. We expect our findings to generalize better to domains that correlate highly with traits like these (e.g., prosocial behavior), compared to domains that are less correlated (e.g., basic perceptual development). Third, our approach is more important for research that focuses on how children *typically* perform in certain tasks (e.g., research aiming to provide normative data). Sampling bias may be less of a concern for research that focuses on competence (e.g., showing that at least some children can demonstrate certain capacities in an ideal context). Finally, pre-consent observations are ethically viable only for public actions, and the complexity and quality of coding may be limited by what can be observed without the dyad’s awareness.

In cases where our approach can be applied, it could benefit the interpretation and generalization of experimental findings in several ways: First, it could reveal correlations between parent–child interactions and children’s behavior, which may help explain the cognitive mechanisms and environmental inputs associated with the observed behavior. Second, it could inform the generalizability of experimental findings to children whose parents did not consent them to participate. Third, it can serve as an empirical base for future research to recruit a more representative sample. By knowing the associations between parental consent and patterns in parent–child interaction, it may be possible to intentionally focus recruitment on parent–child dyads who are likely underrepresented in typical recruitment procedures.

Our findings support and add to the recent concern about persistent sampling biases in psychology broadly (Arnett, 2008; Henrich, Heine, & Norenzayan, 2010), and developmental psychology in particular (LeWinn, Sheridan, Keyes, Hamilton, & McLaughlin, 2017; Nielsen, Haun, Kärtner, & Legare, 2017). We show that in addition to the biases resulted from unrepresentative pools of participants researchers usually recruit from, the process of recruitment itself may also skew the sample. Specifically, we show that variations in parenting practices may directly associate participation with measurements of children’s behavior, which may explain some of the sampling biases associated with indirect factors such as culture, race, and socioeconomic status. Therefore, although existing sampling techniques (such as stratified sampling across sociocultural factors) and analytical tools (such as weighting adjustments) could help in balancing *who* comprises a research sample, understanding *how* findings from a research sample can be generalized or not may require measurements of factors more closely related to children’s behavior, such as patterns of parent–child interactions. One way to do that, as suggested by this study, is to pair experimental studies with naturalistic observations of parent–child interactions.

Our results may also have implications for developmental theories. Many developmental theories are built upon findings from experiments, as experimental designs have advantages in addressing a range of developmental questions, such as depicting developmental trajectories, disentangling causal mechanisms underlying children’s behavior, and testing causal effects of interventions. In typical cases, random assignment of participants across groups removes unwanted systematic differences between groups, so that the effects of age, manipulations, or treatments can be estimated by comparing between-group differences with within-group differences. However, our results have shown that parental consent may bias these comparisons in two ways that random assignment cannot solve: First, it could lead to an underestimation of within-group variations, and thus Type I errors may be underestimated and effect sizes may be overestimated. Second, compared to the general population, children who received consent may be more susceptible or insusceptible to certain manipulations or treatments, therefore biasing the estimation of the between-group differences. Because theories built upon experimental findings often guide real-world practices that apply to the general population, understanding factors and biases associated with nonenrollment is essential when interpreting and applying these findings.

To conclude, this study provides an empirical demonstration that preschool children with and without parental consent to participate in research may behave differently in experiments. In addition, we provided a method that could be used to estimate the biases in experimental results that are related to parental consent.

## ACKNOWLEDGMENTS

We thank Reham Bader, Merna Botros, Milagros Grados, Anishka Jean, and Natasha Patel for help in testing and coding data. We also thank Vanessa LoBue and members of the Child Study Center for helpful feedback on an earlier draft of this manuscript. We thank all participating children and their parents.

## FUNDING INFORMATION

PS, National Science Foundation (http://dx.doi.org/10.13039/501100008982), Award ID: SMA-1640816. EB, National Science Foundation (http://dx.doi.org/10.13039/501100008982), Award ID: ECR-1660885. EB, Jacobs Foundation (http://dx.doi.org/10.13039/501100003986).

## AUTHOR CONTRIBUTIONS

YY: Conceptualization: Supporting; Data curation: Lead; Formal analysis: Lead; Methodology: Lead; Visualization: Lead; Writing—Original Draft: Lead; Writing—Review & Editing: Lead. PS: Conceptualization: Equal; Funding acquisition: Equal; Supervision: Equal; Writing—Review & Editing: Supporting. EB: Conceptualization: Equal; Funding acquisition: Equal; Supervision: Equal; Writing—Review & Editing: Supporting.

## Notes

^1^ The multiple imputation procedure uses a multiple regression model fitted on the participating parents and children to impute the behavior of the not-participating children. The propensity score matching–procedure selects subsamples of the participating dyads who, judging from patterns of parent–child interaction, were unlikely to participate, and data from these subsamples are then used to simulate the behavior of the not-participating children.^2^ Detailed procedures for each phase and the coding scheme for parent–child interactions can be found in the Supplemental Materials C (Yu et al., 2020).^3^ The rationale of using multiple imputation and the technical details of the implementation are provided in the Supplemental Materials I and J (Yu et al., 2020).^4^ The rationale of using propensity score matching and the technical details of the implementation are provided in the Supplemental Materials K and L (Yu et al., 2020).^5^ We thank an anonymous reviewer for suggesting this method of cross-validation.
