# Supplementary material for: Inconvenient Samples: Modeling Biases Related to Parental Consent by Coupling Observational and Experimental Results
Source: Open Mind (Camb). 2020 Mar 1;4:13–24. doi: 10.1162/opmi_a_00031 (PMC7323845; doi:10.1162/opmi_a_00031)
Supplement: Supplementary file 1 [file opmi-04-13-s001.pdf]

Yu, Y., Shafto, P., & Bonawitz, E. (2020). Inconvenient Samples: Modeling Biases Related to Parental Consent by Coupling Observational and Experimental Results. *Open Mind: Discoveries in Cognitive Science*. Supplemental Material. https://doi. org/10.1162/opmi_a_00031

A. Estimating parental non-consent in developmental studies

We circulated a brief survey among lab managers and project leaders through the email list of Cognitive Development Society, a leading society of researchers who conduct experimental research with infants and young children. Responses from 30 researchers indicated an overall consent rate of 50%, and the rate varied across different recruitment methods (consent rate = 37.8% for consent forms sent to children's preschool or school; 50.3% for direct phone call or email to parents; 84.4% for on-site recruitments such as those conducted in science museums).

B. Participants

We set up the study in two sites: an indoor reptile exhibit in a zoo, and an indoor playground. The zoo is in Essex County, NJ, which is one of the nation's most racially diverse counties, and has one of the nation's most unequal economies measured by the Gini Index (U.S. Census Bureau, 2016). The playground is in Middlesex County, NJ, which is overall more affluent, but also has a highly racially diverse population, of which 30% were born outside the U.S. (U.S. Census Bureau, 2016). We chose these two sites to ensure diversity in the population we initially observed. Consents from these two sites were obtained before conducting the study.

Between the two sites, 31 dyads were observed but were not invited for the test because of one of the following: the dyad left before the observation was finished (18), researchers did not get a chance to invite parent because parent was busy (e.g. on the phone) or on the way out

(4), adult accompanying child was not the child's parent (4), parent interrupted researchers during observation (1), child was out of the target age range (2), or child did not speak English (2). The remaining 78 parent-child dyads comprised our "population", including 32 mother-son dyads, 16 mother-daughter dyads, 17 father-son dyads, and 13 father-daughter dyads. According to parental report, children who participated in the test phase were diverse regarding race (51% white, 4% black, 15% Hispanic-Latino, 13% Asian, 17% multi-racial), but most came from middle- or upper-class families (91% of the main caregivers have college diploma or above, 84% of the families have annual household income of $50K or above).

C. Observation phase

Two coders coded 5-minute sessions of parent-child interactions based on the instructions and coding sheet listed below. Using a stopwatch, the coders marked periods of different activities on a timeline (they used separate columns for solitary and dyadic activities, which helped determine the time for the three kinds of activities). The coders also wrote down language from the parent to the child. Parent-child communications were typically not too frequent or fast, so that the coders could at least catch and write down the parent's key words, which could help determine the type of communication (e.g., whether a question is pedagogical or information-seeking) later. For all dyads, two coders agreed on when to start, and both took notes for the same 5 minutes. After the 5 minutes they independently coded from the notes according to the coding instructions, before a third researcher approached the parent to solicit participation. All coders were trained for approximately 5 hours (including discussions of procedure and coding schemes and familiarization of the test sites), and also practiced the coding scheme with at least 5 parent-child dyads before formal data collection. Here are the coding instructions which the

coders used during observation. The superscript numbers in the coding sheet correspond to items in the coding section on the instruction sheet.

Date: Time: Testing site: Participant number:

The party (e.g., mom, dad, older brother, younger sister, infant):

The target parent-child dyad (e.g., mom & younger sister):

Minute	Child's solitary activity (location, behavior, look, companion)[3]	Parent's solitary activity (location, behavior, look, companion)[3]	Parent Child interactions (location, behavior, language, look)[4]
0:00			
0:30			
1:00			
1:30			
2:00			
2:30			
3:00			
3:30			
4:00			
4:30			
5:00			

Child's age:

Did the parent agree to participate in our study?

Note:

Time of parent interacting with child [6]: Time of parent watching child [7]:

Time of parent doing own stuff [8]: # of parent's pedagogical questions [9]:

# of parent's information-seeking questions [10]: # of parent's directions [11]:

# of parent's other comments [12]:

Instructions

Before observation:

1. Make sure you are familiar with the testing site.
2. We focus on children between 3 and 6 years of age. If a child is toddling, he or she might be too young.
3. To reduce bias, follow the first child you saw that may fall in the age range, and try to figure out his/her parent. When possible, code all children in the age range who are present that day (so the result wouldn't be biased because we "pick" certain dyads).
4. To reduce bias, start as soon as figuring out the target parent-child dyad, and don't wait. We want a random (thus representative) 5-minute period.
5. Write down date, testing site, participant number, the party, and the target parent-child dyad.
6. Prepare stopwatch.

Coding:

1. Don't follow the dyad around. Try to sit at a spot that is easy to observe them, move when needed, and avoid eye contact.
2. When parent and child are physically apart, use the first two columns; when they are together (involved in the same activity, talking, or leading/following) use the last column.
3. Write down where they are, what they are doing, and who are with them. Indicate if the parent is looking at the child or the child is looking at the parent.
4. When the parent and child are together, try hard to catch and write down the conversation between parent and child, as well as their interactions.
5. Put down a line when the dyad transit to a different activity/space, for estimating the time they spend on each activity.
6. After 5 min, calculate how much time the parent and child interacted (last column).
7. Calculate how much time parent spent watching the child, taking pictures of the child, looking for the child, but the child was not interacting with the parent (should be a portion of column 2).
8. Calculate how much time the parent spent doing his or her own stuff (should be 5 min minus the above two time periods)
9. Calculate the number of parent's pedagogical questions. Pedagogical question is defined as a question of which the parent knows the answer. Such as "What's that?", "What's the color of the frog?", or "Why wouldn't it turn on?"
10. Calculate the number of parent's information-seeking questions. Information-seeking question is defined as a question of which the parent does not know the answer. Such as "Are you done?" "Which one do you want?", or "Can I try?"
11. Calculate the number of parent's directions towards the child, such as "Let's go", "Come on, you can do it!", "You can play 2 more games".
12. Calculate the number of parent's other comments towards the child, such as "That is cool!", "It's gonna be okay", "Good boy".

Soliciting participation:

1. A third experimenter who is blind of the coding will approach the parent. He will say "I am a postdoctoral researcher from Rutgers University, and I am from a lab that studies how children learn and explore. Today we brought a study with us that aims to investigate how children explore a novel object. It is really fun and will only take about 5 minutes. We will also give you a $5 coupon for your next visit here if you agree to participate. We will need to videotape your child during the experiment, but these videos will only be viewed by research assistants and will never be shared. Would you be interested to let your child participate?"
2. If the parent agree or defer to his/her child, write "yes" for "did the parent agree to participate in our study?", even if the child refuse to play.
3. If both parent and child agree to play, lead them to the place where you set up, let parent sign the consent form and video consent, and put participation number (e.g., PCI16) on the consent form.
4. If the child turns out to be too young or too old, mark it in the "notes" and still play with him/her without video.
5. Turn on camera if you have video consent, and follow the PQ script (pedagogical question condition).
6. Thank parent and child for participation.

D. Recruitment phase

The recruitment procedure followed a script which resembled that of a typical developmental experiment: The researcher started with a brief self-introduction, then described the research as a study of how children learn and explore a novel toy, then briefly explained the consent form, and finally asked if the parent would be interested to have his or her child participate in the test. For parents who had multiple children with them, we specifically asked for the child who had been observed. Of the 59 parents who agreed, 11 children did not participate in the test (10 did not provide assent and 1 did not understand English), and the video was missing for one additional child, so data from the test phase were available for 47 children, who comprised the "participating" group (age 3.0y to 6.3y, 27 were recruited from the zoo and 20 from the playground).

E. Test phase

Parents and children who agreed to participate were led to a corner of the zoo exhibit or a separate room in the indoor playground, where the test was conducted by the recruiter (acting as an experimenter) and one of the coders (acting as a confederate). We used a novel toy of approximately 14" × 7.5" × 14.5" in the test. In addition to several inert properties, the toy had five functional parts: a tower that lit up when a button was pushed, a knob that produced a squeaking sound when squeezed, a lady bug pin light that flashed in three different patterns when pushed, a flower magnet that moved between three different places on the toy, and a turtle hidden in a pipe that was visible through a magnifying window. During the test, the child sat at a table opposite the experimenter and the confederate. The toy was initially hidden out of sight. The experimenter first said that she knew about the toy and the confederate did not, and asked the confederate to bring out the toy. After the confederate brought out the toy and handed it over to the experimenter, the experimenter then asked a pedagogical question to the child, "I'm asking

you to think about: What does this button do?", while pointing to the button on the tower without activating it. Then she told the child it was his or her turn to play with the toy, and to let the researchers know when he or she was done. The test ended when the child stopped playing and signaled the researchers, and a sticker was presented as a reward. The whole phase was video recorded.

F. Inter-rater reliability

The inter-rater reliability agreement was high for all measurements: total time playing: $r = .98$; activating target function: Cohen's $\kappa = 1$ for both total time and first minute; number of non-target functions activated: Cohen's $\kappa = .81$ (total time) and $\kappa = .75$ (first minute); number of unique actions performed: $r = .79$ (total time) and $r = .92$ (first minute).

G. Detailed analysis of cross-site comparisons

The proportion of parents who agreed to participate did not differ significantly across sites—playground: 25 agreed, 12 refused (68% vs. 32%); zoo: 34 agreed, 7 refused (83% vs. 17%); Fisher's exact $p = .19$.

Test data was available for 47 children ranging from 3.0y to 6.3y, of which 27 were recruited from the zoo and 20 from the playground. Children from the two sites did not differ with regard to the activation of target and other functions, or the number of unique actions they performed on the toy, ts < 1.4, ps > .1. However, there was a trend of children playing longer with the toy in the playground than in the zoo, $M_{zoo} = 189$s, $M_{playground} = 132$s, $t(29.4) = 1.83$, $p = .078$, $d = 0.58$. When comparing these results with previous experiments we conducted in preschools ($n = 30$, age range = 4.0y to 6.0y) using the exact same protocol (Yu et al., under review), none of children's response measurements differed significantly across the three sites, Fs < 2.2, ps > .1.

Parent-child interactions varied both across sites and within sites: Compared to dyads in the playground, dyads in the zoo spent more time on dyadic activities, and less time on supervised (but not dyadic) activities or unsupervised activities, ts > 2.6, ps < .01. Parents also asked more pedagogical and information seeking questions, and said more statements in the zoo than in the playground, ts > 3.4, ps < .001. The difference in parents' commands toward children was marginally significant, $t(67.7) = 1.75$, $p = .09$. These results suggest that the test site needs to be considered when interpreting parent-child interactions. Therefore, test site had been entered as a control variable for all further analyses. We also observed large within-site variations: For all measurements, standard deviations were higher than 1/3 of the mean for both the zoo and the playground. These variations suggest that the population we observed was diverse with regard to parent-child interactions, which serves as a basis for further correlational analyses.

H. Detailed analysis of correlations between parent-child interaction and children's behavior during test

When looking at individual measures of exploratory learning, children of parents who spent more time watching and following them were less likely to discover the target function during the first minute of play, $r(42) = -.36$, $p = .018$. At the same time, children whose parents asked more pedagogical questions discovered more other functions of the toy, $r(42) = .32$, $p = .032$, and also performed more unique actions during the first minute of play, $r(42) = .30$, $p = .048$.

I. Rationale in applying model-based multiple imputation

Multiple imputation is the recommended tool to predict missing data when missingness depended on other observed variables, but not the missing variable itself (1). In our case, because missingness was a result of the parent's decision, it was associated with patterns observed in parent-child interactions (as shown in the logistic regression), but not directly associated with

children's behavior in the test. Therefore, multiple imputation is suitable to simulate behavior for the not-consented children.

J. Step-by-step description of applying model-based multiple imputation

The model we used for multiple imputation was a stochastic regression model. The computation was implemented using the Multiple Imputation module of IBM SPSS 22.

Step 1. The seven observational measurements (3 on the lengths of different types of parent-child interactions, and 4 on the frequency of parent-child communication) were used to model the seven test measurements, based on data from the participated group. Among the seven test measurements, two (activation of target function, in total time and in first minute) were imputed as binary variables, whereas all other test measurements were imputed as continuous variables. For each test measurement, a logistic regression model (for binary variables) or a linear regression model (for continuous variables) was fitted on the data from the participating group.

Step 2. For each test measurement, from the fitted model, posterior distributions were computed for the 8 parameters in the logistic regression model (intercept and coefficients for the 7 observational measurements) or the 9 parameters in the linear regression model (intercept, coefficients for the 7 observational measurements, and the residual variance).

Step 3. Then the test measurements for the not-participating group were imputed for m = 100 runs. For each run, a new set of parameters were randomly drawn from their respective posterior distributions, and were used to compute the expected values plus random errors for each child in the not-participating group. The means and standard deviations of the not-participating group and of the whole population were then calculated for this test measurement. The procedure is then repeated for the remaining runs of simulations, and the distributions of the means and standard deviations are shown in Table 2.

Step 4. Step 2 and 3 were then repeated for all other test measurements.

K. Rationale in applying propensity score matching

Propensity score matching (PSM) was used to select subsamples of the participated group that matched the not-participated group in both size and probability to participate. PSM is typically used to estimate the effect of a treatment while controlling for the effect of potential confounders that predict receiving the treatment. Here we consider parental consent as "treatment", so PSM can help find subsamples of children that, judging from the way their parents interacted with them during observation, were not likely to participate in research.

L. Step-by-step description of applying propensity score matching

Propensity score matching was implemented with R 3.2.3.

Step 1. Propensity scores were calculated by fitting a logistic regression model on both the participating and not-participating parent-child dyads. The dependent variable was participation in research (1 for participating, 0 for not-participating), and the 11 independent variables included 4 variables on the gender and composition of the dyad, 3 variables on the lengths of different types of parent-child interactions, and 4 variables on the frequency of parent-child communications (see Table 1 in the main text for the list of all variables). This model resulted in a propensity score for each individual parent-child dyad. The range of the score is from 0 to 1, and a higher score means judging from all independent variables, the dyad is likely to participate in research. The overall average of propensity scores is higher for the participating group than the not-participating group, yet because of individual variations it is still possible to pick parent-child dyads from the participating group who has low propensity scores, which served as the basis for matching.

Step 2. Using group-level non-bipartate matching (sampling with replacement), and a caliper (tolerance on propensity score distance) of 0.2 standard deviations, we repeatedly drew random subsamples of 19 children from the participating group until the average propensity score approaches that of the not-participating group. This was achieved when the propensity score difference (after logit transformation) between the random subsample and the not-participating group was below the caliper.

Step 3. We recorded the subsample and calculated the means and standard deviations of all test measurements based on the subsample.

Step 4. To be consistent with the bootstrapping and multiple imputation groups, subsamples were drawn for m = 100 runs, and the means and standard deviations of the test measurements were recorded for each subsample. The distributions of these means and standard deviations were then used to verify the results from the multiple imputation, and are shown in Figure 2 and S1.

Table S1

Partial correlations between children's test measurements and dyads' observational measurements, after controlling for testing site and children's age

	Exploration variability	Exploration efficiency	Total play time	Target function		Other functions		Unique actions	
				total[1]	1min[2]	total	1min	total	1min
Parent gender	.01	.07	.05	.17	.04	-.01	.01	-.17	-.20
Child gender	-0.16	-.08	-.03	.13	.07	.11	.03	.14	.11
Other adult	.23	.24	-.02	.18	.25	.21	.22	.18	.10
Other child	-.08	.05	-.07	.14	.22	-.13	-.04	-.18	-.05
Dyadic	.15	**.35***	-.18	.12	.29	.15	.27	.09	.27
Supervised	-.17	-.28	.18	-.19	**-.36***	-.18	-.22	-.05	-.11
Unsupervised	.00	-0.21	.13	.01	-.05	.03	-.16	-.03	-.27
PQ[3]	**.30***	**.35***	-.12	.25	.26	**.32***	.26	.17	**.30***
ISQ[4]	.07	.18	-.03	.12	.22	.15	.22	-.09	-.01
Statements	.11	.11	-.04	.16	.26	.16	.02	.09	.17
Commands	.16	.19	.21	.20	.23	-.04	.04	.13	.01

Notes. 1. total = During the entire period of play

2. 1min = During the first minute of play

3. PQ = Pedagogical questions

4. ISQ = Information-seeking questions

*$p < .05$.

Table S2

Logistic regression model predicting parents' participation

	B	*SE* (*B*)	*β*	Wald[1]
Test site (zoo - playground)	0.20	0.85	.031	0.06
Parent gender (male - female)	0.82	0.72	.109	1.30
Child gender (male - female)	1.47	0.69	.185	4.55*
Other adult (present - absent)	0.50	0.70	.065	0.51
Other child (present - absent)	0.49	0.61	.055	0.66
Dyadic activities[2]	-0.11	0.30	-.006	0.13
Supervised activities[2]	0.44	0.31	.025	1.98
Pedagogical questions	1.49	0.77	.210	3.71*
Information-seeking questions	-0.07	0.32	-.004	0.06
Statements	-0.05	0.21	-.002	0.06
Commands	0.56	0.32	.033	3.10

Notes. 1. The Wald chi-square test was used to test whether the coefficients were significantly different from 0.

2. The three measurements for the length of parent-child interactions added up to a constant value (5 min), so only two were included in the model to avoid multicollinearity.

$^{*}p < .05$.

Table S3

Summary of results from the model-based multiple imputation

		Partici-pating	Not-participating[1]		Combined[1]		*d*
			Estimate	95% CI	Estimate	95% CI	
Total play time	*M*	156.3	161.0	155.7-166.3	157.7	156.1-159.2	.05
	SD[2]	101.3	107.7**	103.4-111.9	103.1**	101.9-104.4	—
Activation of target function (total)	*M*	.660	.567***	.541-.594	.633***	.625-.640	.19
	SD	.479	.490**	.483-.496	.484***	.482-.486	—
Activation of target function (1min)	*M*	.610	.506***	.479-.533	.585***	.577-.593	.23
	SD	.491	.494	.488-.50	.495***	.494-.496	—
Number of non-target functions discovered (total)	*M*	1.74	1.70	1.63-1.77	1.73	1.71-1.75	.04
	SD	1.13	1.26***	1.21-1.31	1.17***	1.16-1.19	—
Number of non-target functions discovered (1min)	*M*	.830	.744***	.697-.790	.805***	.792-.818	.10
	SD	.816	.905***	.867-.942	.844***	.832-.855	—
Number of unique actions performed (total)	*M*	6.23	5.94**	5.72-6.15	6.15**	6.09-6.21	.08
	SD	3.49	4.05***	3.88-4.21	3.66***	3.61-3.72	—
Number of unique actions performed (1min)	*M*	3.85	3.40***	3.26-3.53	3.72***	3.68-3.76	.18
	SD	2.42	2.61**	2.49-2.72	2.49***	2.45-2.52	—

Notes. 1. For parameters (*M* and *SD*) of each test measurement, we estimated their distributions for the not-participating group (n = 19) and for the participating and not-participating group combined (n = 66) over the 100 runs of imputations, and compared the distributions to the parameters from the participating group (n = 47).

2. *SD* = sample standard deviation. For the not-participating and combined groups, the estimates of *SD*s were calculated by taking the quadratic means of *SD*s over multiple runs, instead of taking the arithmetic means. This is because while SD^2 is an unbiased estimator for population variance, *SD* is a biased estimator for population standard deviation. Similarly, 95% CIs were calculated by taking the squared root of 95% CIs for sample variances across multiple runs.

$^{**}p < .01$, $^{***}p < .01$. These asterisks denote significant departures of the estimated parameters from the observed parameters over 100 runs of imputation.

Table S4

Summary of results from cross validation for model-based multiple imputation

		Remain-ing	Left-out			
			Observed	Simulated	95% CI	*d*
Total play time	*M*	157.5	154.3	153.0	146.6-159.5	.01
	SD	100.4	105.6	105.5	101.9-109.0	—
Activation of target function (total)	*M*	.655	.667	.672	.644-.701	.01
	SD	.484	.485	.478	.463-.492	—
Activation of target function (1min)	*M*	.621	.611	.634	.603-.666	.05
	SD	.494	.502	.513	.495-.532	—
Number of non-target functions discovered (total)	*M*	1.72	1.78	1.79	1.72-1.86	.01
	SD	1.07	1.26	1.28	1.22-1.33	—
Number of non-target functions discovered (1min)	*M*	.828	.833	.862	.823-.902	.03
	SD	.759	.824*	.784	.753-.815	—
Number of unique actions performed (total)	*M*	6.27	6.38	6.22	6.04-6.41	.05
	SD	3.57	3.45	3.50	3.38-3.61	—
Number of unique actions performed (1min)	*M*	3.90	3.78*	3.92	3.80-4.03	.05
	SD	2.34	2.42	2.38	2.30-2.47	—

Notes. $^{*}p < .05$. These asterisks denote significant departures of the simulated parameters from the observed parameters over 100 runs of imputation.

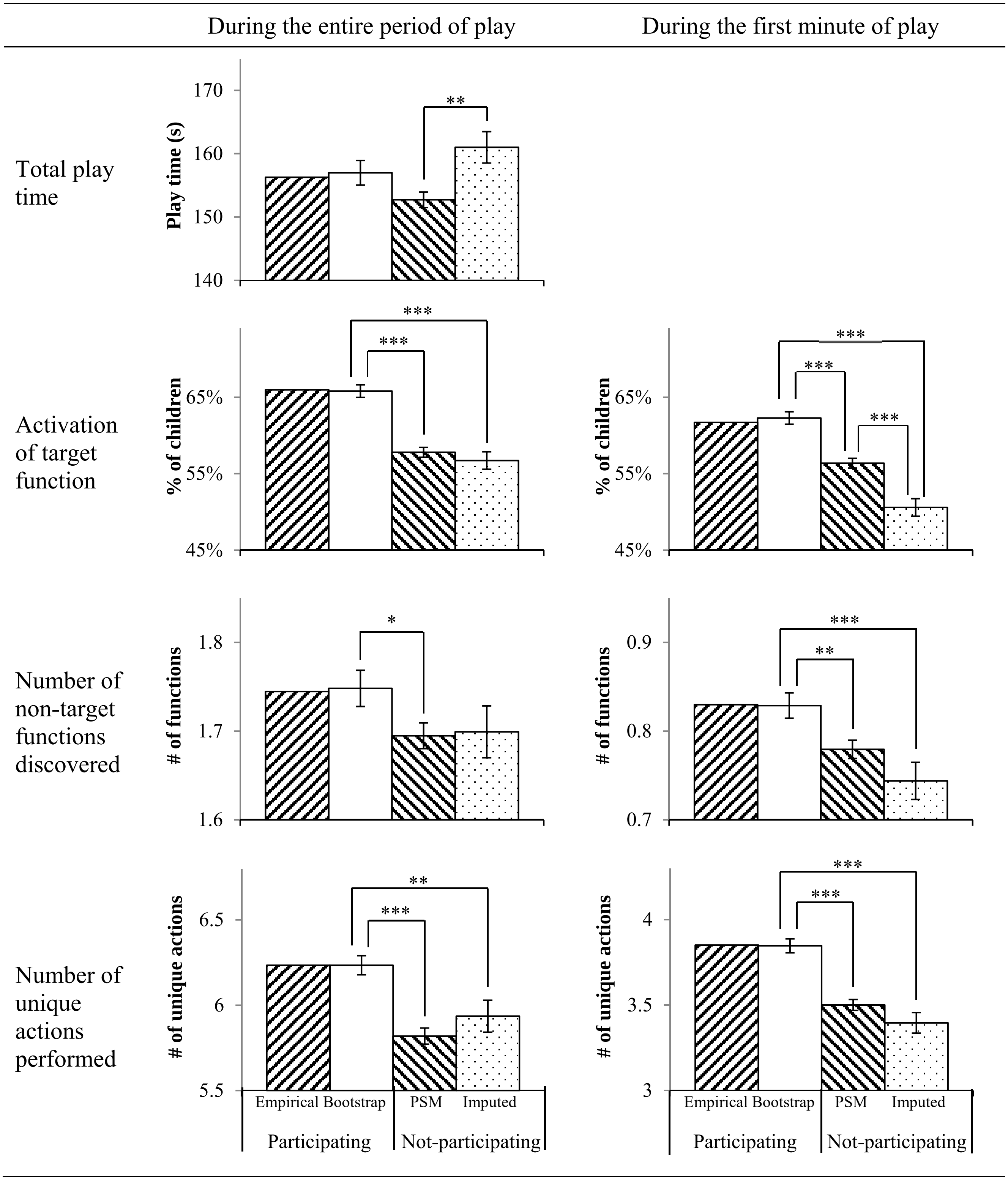

During the entire period of play
During the first minute of play
Total play time
Play time (s)
140
150
160
170
**
Activation of target function
% of children
45%
55%
65%
***
Number of non-target functions discovered
# of functions
1.6
1.7
1.8
*
0.7
0.8
0.9
**
Number of unique actions performed
# of unique actions
5.5
6
6.5
3
3.5
4
Empirical
Bootstrap
PSM
Imputed
Participating
Not-participating

Figure S1. Comparisons of the estimated group means between the participating group and the simulated not-participating group on all 7 test measurements. Empirical = children who actually participated in the test phase ($n = 47$); Bootstrap = randomly selected subsamples of the participating group that matches the not-participating group in size ($n = 19$); PSM = subsamples of the participating group that matches the not-participating group in size and propensity score ($n = 19$); Imputed = simulations of the not-participating group using a model-based multiple imputation procedure ($n = 19$). To examine the robustness of simulation results, the Bootstrap, PSM, and Imputed groups were resampled for $m = 100$ runs, and the standard error across all runs are shown in the figure as the error bars. For 5 out of the 7 measurements, the two ways of simulating the not-participating group (PSM and Imputation) resulted in group means that were not significantly different from each other. In contrast, for all measurements except for total play time, the estimated group means of the not-participating group were significantly lower than that of the participating group, indicating an overestimation of children's test performance by not considering those who were not consented to participate. *$p < .05$; **$p < .01$; ***$p < .001$

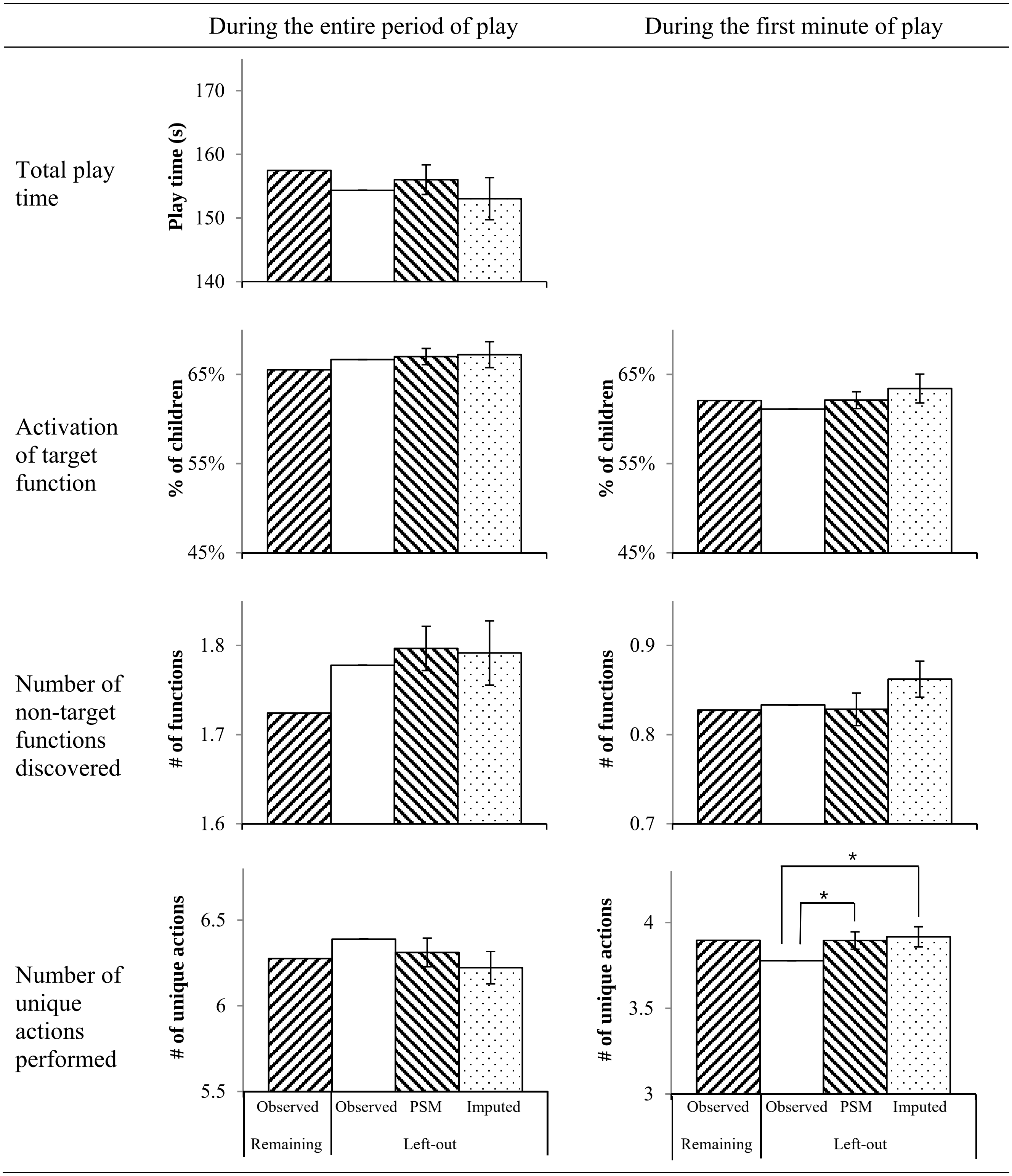
During the entire period of play
During the first minute of play
Total play time
Activation of target function
Number of non-target functions discovered
Number of unique actions performed
Play time (s)
% of children
# of functions
# of unique actions
170
160
150
140
65%
55%
45%
1.8
1.7
1.6
0.9
0.8
0.7
6.5
6
5.5
4
3.5
3
*
*
Observed
Observed
PSM
Imputed
Remaining
Left-out

Figure S2. Comparisons of the simulated and observed data from the cross validation. Left-out = half of the boys (*n* = 18) whose data was falsely left out for cross validation; Remaining = the remining children (*n* = 29); Observed = actual data collected in the test; PSM = subsamples of the remaining group that matches the left-out group in size and propensity score (*n* = 18); Imputed = simulations of the left-out group using a model-based multiple imputation procedure (*n* = 18). Similar to the main analysis between participating and not-participating groups, PSM and Imputed groups were resampled for *m* = 100 runs, and the standard error across all runs are shown in the figure as the error bars. The two ways of simulation (PSM and Imputation) resulted in group means that were not significantly different from each other for all test measurement, and for 6 out of 7 measurements they were not significantly different from the observed group means. $^*p < .05$.

References

Sinharay, S., Stern, H. S., & Russell, D. (2001). The use of multiple imputation for the analysis of missing data. *Psychological Methods, 6*(4), 317.

U.S. Census Bureau. (2016). 2011-2015 American community survey. Retrieved from factfinder.census.gov
